# Orientation and navigation in *Bufo bufo*: a quest for repeatability of arena experiments

**DOI:** 10.3897/herpetozoa.33.e52854

**Published:** 2020-08-14

**Authors:** Markus Pail, Lukas Landler, Günter Gollmann

**Affiliations:** 1 University of Vienna, Department of Evolutionary Biology, Althanstraße 14, 1090 Vienna, Austria; 2 Institute of Zoology, University of Natural Resources and Life Sciences, Gregor-Mendel-Straße 33/1, 1180 Vienna, Austria

**Keywords:** Amphibia, behavioural ecology, Bufonidae, direction following, MANOVA, migration

## Abstract

Research on navigation in animals is hampered by conflicting results and failed replications. In order to assess the generality of previous results, male *Bufo bufo* were collected during their breeding migration and translocated to two testing sites, 2.4 and 2.9 km away, respectively, from their breeding pond in the north of Vienna (Austria). There each toad was tested twice for orientation responses in a circular arena, on the night of collection and four days later. On the first test day, the toads showed significant axial orientation along their individual former migration direction. On the second test day, no significant homeward orientation was detected. Both results accord with findings of previous experiments with toads from another population. We analysed the potential influence of environmental factors (temperature, cloud cover and lunar cycle) on toad orientations using a MANOVA approach. Although cloud cover and lunar cycle had small effects on the second test day, they could not explain the absence of homeward orientation. The absence of homing responses in these tests may be either caused by the absence of navigational capabilities of toads beyond their home ranges, or by inadequacies of the applied method. To resolve this question, tracking of freely moving toads should have greater potential than the use of arena experiments.

## Introduction

The question of how animals navigate has been investigated in numerous species over many decades (Able 1995; Sinsch 2006; Chernetsov 2016). While progress has been made in the understanding of the neuronal basis of small-scale spatial navigation (Moser et al. 2008; Cullen and Taube 2017), other fundamental problems are still poorly understood. It is still unclear how and, in some cases, if animals can home after displacement to unfamiliar sites, without direct contact to the goal. Such ability has been termed ‘true navigation’ and has been shown for newts, spiny lobsters and migratory birds (Phillips et al. 1995; Boles and Lohmann 2003; Kishkinev et al. 2015). Recently, it has been argued that true navigation might not be a general ability of animals, but rather a specialized sense of certain taxa (Sinsch and Kirst 2015). This discussion is related to the question of the underlying sense that would allow such abilities. Two sensory systems have been suggested to provide positional information: the magnetic (Freake et al. 2006; Lohmann et al. 2007) and the olfactory sense (Gagliardo 2013). The magnetic intensity and inclination decrease from the poles to the equator and can therefore provide a spatial grid (Freake et al. 2006). Odours, as well as their ratios, can vary predictably between different locations and might be extrapolated beyond familiar areas (Wallraff 2004). The latter hypothesis has never been tested for ground-dwelling animals, and it has been argued that odours might be unreliable for amphibians (Diego-Rasilla et al. 2008). Orientation research, however, is poised by numerous conflicting results and failed replications (Hein et al. 2011; Edelman et al. 2015; Landler and Siegel 2016). Therefore, replication of published studies is necessary to assess the robustness of previous findings (Nimpf and Keays 2020).

While the function of the olfactory system is well understood, the mechanisms underlying magneto-sensation are still debated. Currently, there are three main hypotheses of magnetoreception: 1) the light-dependent mechanism, based on the formation of spin-correlated radical pairs (Hore and Mouritsen 2016); 2) magnetite-based magnetoreception, based on small clusters of magnetite which might be tethered to ion channels (Kirschvink et al. 2001); 3) induction-based magnetoreception: Changes in the magnetic field may induce electric currents in the inner ear, as it has been proposed for pigeons (Nimpf et al. 2019). Studies in newts, but also anuran species, showed a wavelength dependency of magnetic compass orientation, which is suggestive for a light-dependent magnetic compass mechanism, possibly located in the pineal organ (Deutschlander et al. 1999; Diego-Rasilla et al. 2013). In contrast, positional information might be derived from a magnetite-based magnetoreception system, however, in amphibians this has so far only been investigated in red spotted newts (Brassart et al. 1999) and the location of such receptors remains elusive.

One might wonder why amphibians should possess such elaborate spatial capabilities at all, as they are usually regarded as small and slow-moving animals. Nevertheless, they can accomplish quite surprising spatial tasks and home from large distances, compared to their size. The common view might need some rethinking. Typical amphibian home ranges might only cover a few hundred meters, but some (e.g. red-bellied newts and water frogs) have been shown to home from up to 4 km and even 15 km (Twitty et al. 1964; Tunner and Kárpáti 1997). In addition to the above mentioned olfactory, as well as magnetic sense, also acoustic and visual cues have been shown to be involved in amphibian navigation (Grubb 1976; Diego-Rasilla and Luengo 2004, 2007; Madden and Jehle 2016). Such multimodal sensory integration and flexibility of the cues used may allow good homing performance observed in some amphibian species (Adler 1980; Pašukonis et al. 2013).

From all anuran species the European common toad (*Bufo bufo*) is arguably the best investigated one in terms of its homing abilities. To quickly summarize the cornerstones of previous common toad migration studies: They have a tightly controlled and highly active (explosive) breeding migration (Jungfer 1943; Gittings 1983; Sinsch 1988), high site fidelity (Reading et al. 1991) and they are able to find back to their breeding ponds after experimental displacement (Heusser 1964). In a study by Sinsch (1987) seven out of ten toads homed successfully after displacement of 3 km, however, they needed up to 3 days to re-orient in the homeward direction. A variety of spatial references have been shown to be used in common toad migration, these include magnetic, acoustic, olfactory and visual cues (Heusser 1960; Sinsch 1987; Höglund and Robertson 1988; Buck-Dobrick 2001). Despite all the studies investigating homing in common toads, it is unclear whether they can home from unfamiliar sites (‘true navigation’). In fact, also in other species it has been questioned if ‘true navigation’ represents a general ability of amphibians or if it might be a restricted phenomenon only present in a few species or even populations (Pašukonis et al. 2014; Sinsch and Kirst 2015).

In earlier experiments, we investigated whether we could elicit navigational responses in the common toad (Landler and Gollmann 2011, 2012; Landler et al. 2016). In the first experiments we collected toads, which were on the way to their spawning pond in the west of Vienna and translocated them 2.5 km. Toads were then tested in an outside arena in the natural magnetic field or in an altered magnetic field. While the magnetic field influenced the orientation behaviour of the toads, they did not orient themselves towards the spawning area, instead they followed their former migratory direction. Such behaviour has been termed d-axis orientation (Endler 1970). Direction following has also been shown by Shakhparonov and Ogurtsov (2016) in a T-maze assay using marsh frogs (*Pelophylax ridibundus*); here frogs followed their migration direction after being placed in a T-maze and magnetic field changes led to changes in orientation preference.

Also, in follow-up experiments where we translocated toads from the same migration route 2.1 km to an indoor testing set-up, toads showed direction following behaviour when tested at the same night. However, when we left the toads at the testing site for 3 days, presumably enough time for the toads to update their internal map, they oriented randomly (Landler et al. 2016). We concluded that testing the toads in an indoor arena and thereby depriving them from a variety of environmental cues, such as celestial and olfactory cues, might have contributed to such results. Alternatively, toads might not possess ‘true’ navigational abilities.

For the present study, we collected toads migrating to another pond in the north of Vienna and tested them in the same arena at two different sites, located approximately 120° apart with respect to the pond. Our aims were twofold: first, to examine whether we could replicate the direction following behaviour immediately after collection; second, whether we could elicit a ‘true’ navigation response in an outdoor situation, after the toads had been kept at the testing site for 4 days, presumably enough time to update their positional information.

## Methods

### Experimental animals

**Figure 1:**
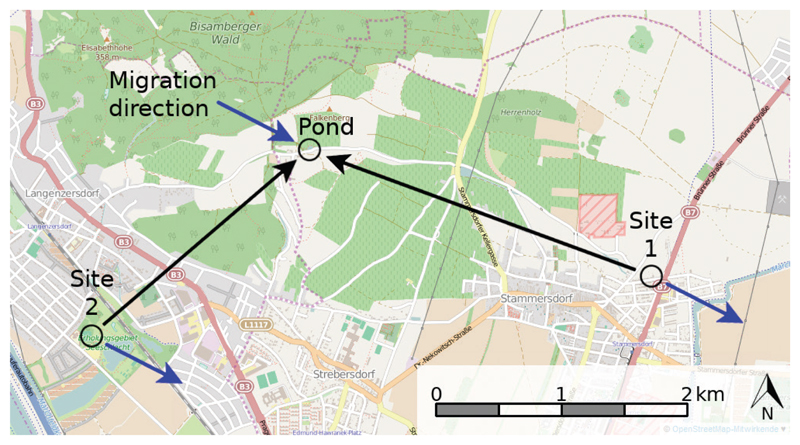
Map of the breeding pond and the two test locations. Toads were collected nearby the breeding pond and translocated to one of the two testing locations. The blue arrows indicate the mean migration direction (all toads were collected in the northwest of the pond), the black arrows the direction of possible homeward orientation.

Male toads (*B. bufo*) were collected during their spawning migration close to their breeding pond (on Bisamberg, Vienna, Austria, 48.31294N, 16.38474E) and translocated to one of two testing sites; ‘site 1’, 2.9 km away (backyard in Floridsdorf, Vienna, Austria, 48.30458N, 16.42174E, homeward direction: 292°), and ‘site 2’ (Seeschlacht in Langenzersdorf, Lower Austria, Austria, 48.29834N, 16.36147E, homeward direction: 45°), 2.4 km away (Fig. 1). For each toad the migration direction was noted in a field protocol, in order to test for orientation relative to the migration direction. We placed a wooden stick next to the toad along its axis and measured the direction to the next 5° interval using a compass. The average body mass of the toads was 37 g and the average snout-vent length was 71 mm.

### Experimental procedure

Experiments took place from 10 to 23 April 2013 from dusk to approximately midnight. Mean testing temperature was 10 °C (SD: 4 °C) and mean cloud cover was 30% (SD: 40%). Following collection, toads were placed in uncovered plastic buckets and transported to one of the two testing sites by car. Testing began immediately after arriving at the testing site; the testing order was identical to the order of collection. Before, and after testing, plastic buckets with toads were placed 10 to 20 m away from the testing rig. Toads were tested in a visually symmetrical circular arena (diameter: 121 cm, height: 60 cm), which had been used in previous studies (Landler and Gollmann 2011; Landler et al. 2016). Inside the arena, toads were able to see the sky, but the arena wall blocked the view of the horizon. Each toad was placed singly in the centre of the arena under a release device (clay pot, diameter: 20 cm). After 4 minutes the release device was lifted with a string from outside the arena without disturbing the animal. Each toad was given 10 minutes to reach the wall of the arena; toads that failed to reach the wall in time were excluded from further analyses. Between trials the arena floor was wiped dry in order to remove potential chemical cues. For each trial the temperature was recorded, and cloud cover was estimated in percent.

After this first day of experiments, animals were held for 4 days at the same location in the plastic buckets; the toads were kept wet the whole time, in order to prevent desiccation. On day 4 toads were tested again (second day of experiments), in order to test for a homing response (‘true navigation’ sensu Phillips et al. (1995)) using the same experimental procedure. All toads collected in an evening were tested at the same site. Every two days the experiments switched to the other testing site. After the experiments, all toads were released at the breeding pond.

Infrared lights and an infrared camera (Panasonic NV-DS28EG) were used to record the trials. From the recorded videos screenshots were taken using the VLC media player 2.0.3 and then the image manipulating software GIMP 2.8. An inner radius was used (85% of the whole arena diameter) to determine the directional preference of each toad. Earlier experiments had shown that toads tend to follow the wall when being close to it, without immediately touching it, leading to a less clustered orientation. The direction for each toad was defined as the direction where the toad crossed the 85% criterion circle and measured to the nearest 5°.

### Statistical analysis

Orientation data for each of the sites and test days were analysed using standard circular statistics (Landler et al. 2018). The Rayleigh-test with specified mean direction (V-test) was used to test for significant orientation along the expected direction (d-axis or homeward). We also tested for axial orientation as such responses had been reported in similar experimental set-ups. In order to test for bimodal orientation individual angles were doubled and resulting mean angles were reduced to modulo 360°. All circular statistics were performed in R (R Development Core Team 2012) using the package *circular* (Agostinelli and Lund 2013) and adapted functions (see Suppl. material 1: R-script 1 for the R code which was used for the analysis and plots. Together with the Suppl. material 2: Table S1, Suppl. material 3: Table S2 and Suppl. material 4: R-script 2, respectively, this can be used to reproduce our results).

In order to test potential influences of weather or lunar cycle on orientation we performed a MANOVA (using the function *lm* together with *Manova* from the package *car* (Fox and Weisberg 2019)). For the two response variables we used the x and y component of the toad orientations with respect to geographic north. This was done using trigonometric functions, i. e. calculating the sine and cosine of the orientations in radians (see Pewsey et al. (2013) for using trigonometric functions in linear models). The lunar cycle was calculated using the *getMoonIl-lumination* function from the package *suncalc* (Thieurmel and Elmarhraoui 2019). The lunar cycle is another circular variable and we therefore split it in the x and y component using the same approach as above. We also used temperature, cloud cover and the testing location as explanatory variables. In order to avoid over-fitting, we made use of an automated AIC based model selection using the function *mStep* from the package *qtlmt* (Cheng 2017). We derived effect sizes (eta^2^) for all terms included in the selected model using the function *etasq* from the package *heplots* (Fox et al. 2018).

Circular plots were generated using an adapted *plot. circular* function derived from the package *circular* (see R Code in Suppl. material 1: R-script 1). Bootstrap confidence intervals were calculated using the function *mle.vonmises.bootstrap.ci* from the same package.

## Results

**Figure 2:**
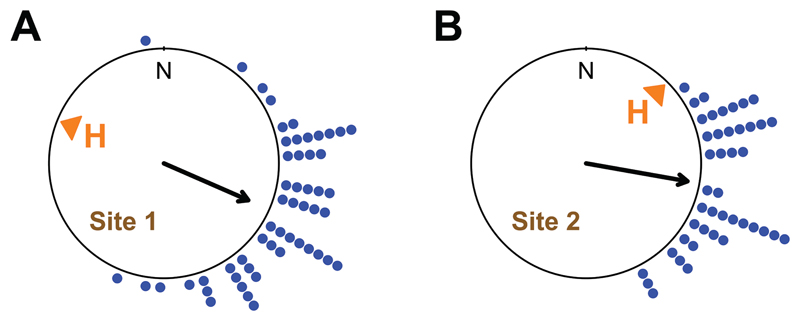
Directions of measured migration directions (d-axis) for each toad for site 1 **(A)** and site 2 **(B).** The arrows represent the mean vectors of the distributions (radius of the circle corresponds to a vector length of 1). Each dot represents the orientation of a single toad.

Out of 116 toads 96 were successfully tested and reached the arena wall in time. The individual d-axis directions of the toads tested at both sites were tightly clustered around 110° with respect to north (Fig. 2).

**Figure 3:**
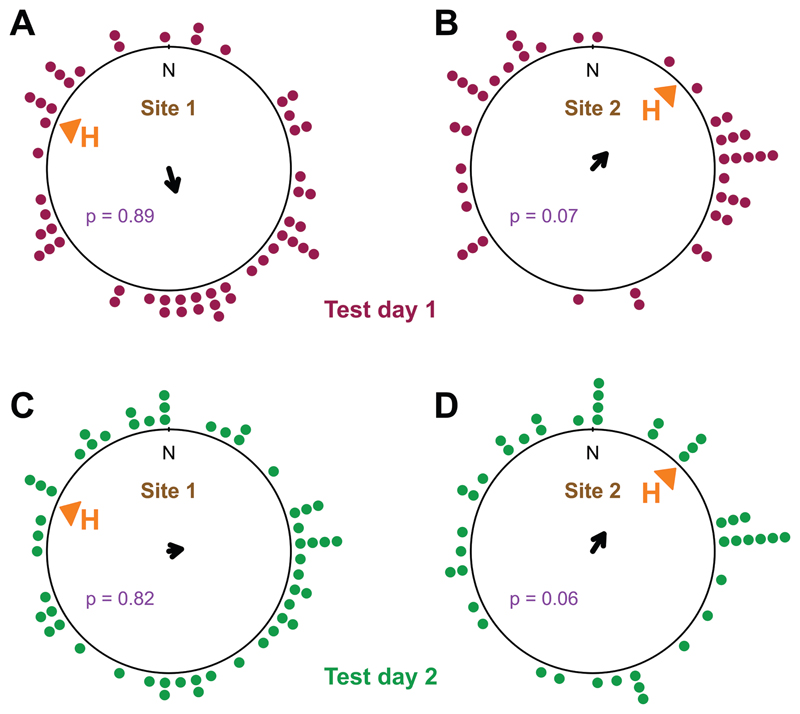
Toad orientation on the first test day at site 1 **(A)** and site 2 **(B)** and the second test day at site 1 **(C)** and site 2 **(D),** relative to geographic north. The arrows represent the mean vectors of the distributions (radius of the circle corresponds to a vector length of 1), none of distributions reached significance (p-values (p) shown in the plots). Each dot represents the orientation of a single toad.

On both test days and sites toads oriented randomly, when analysed towards geographic north (Fig. 3).

**Figure 4:**
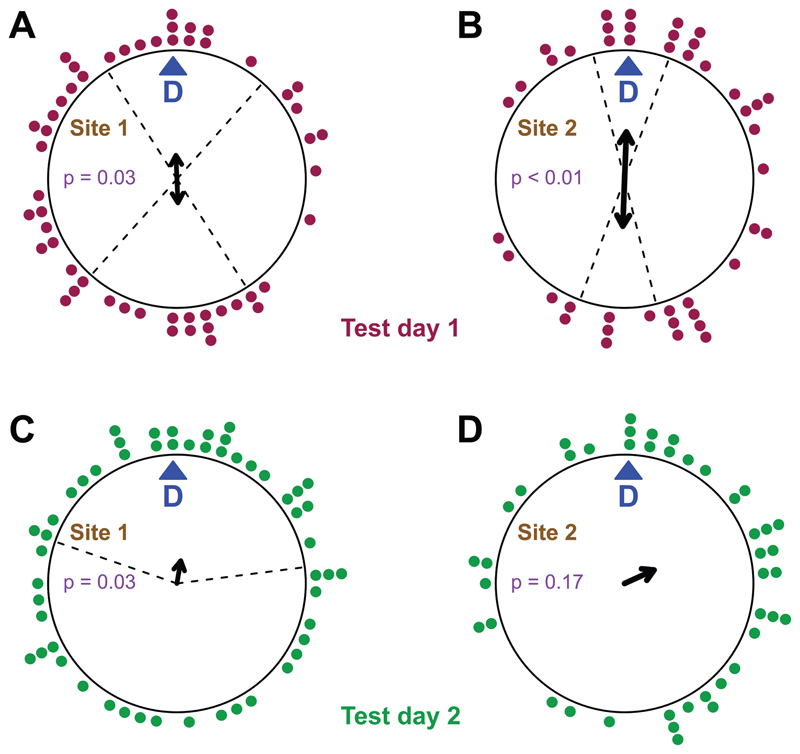
Toad orientation on the first test day analysed for site 1 **(A)** and site 2 **(B)** and the second test day for site 1 **(C)** and site 2 **(D),** relative to the d-axis. The arrows represent the mean vectors (the circle’s diameter equals r = 1, doubled headed arrows in case of axial orientation). P-values (p) are given in each plot. Dotted lines indicate bootstrap 95% confidence intervals for significant orientations. Each dot represents the orientation of a single toad.

In contrast, when analysed relative to the individual former migration direction, toads showed significant axial orientation along the expected direction at the evening of collection. Four days later toads showed weakly significant unimodal orientation towards the d-axis direction at site 1 but no significant orientation at site 2 (Fig. 4).

Weather and the lunar cycle had only minor effects on the orientation. On the first test day the selected model included cloud cover and location, however, none of the two reached significance and the effect size (eta^2^) was well below 0.1 for both factors (Table 1).

Also, on the second test day the effect size for all factors was below 0.1, however, cloud cover and the cosine of the lunar cycle reached significance (Table 2). Interestingly, the location (i.e. site of testing) did not significantly influence orientations, although the homeward direction differed between sites.

## Discussion

In the present experiment we confirmed direction following (d-axis) orientation behaviour in toads tested at the evening of collection (Fig. 4A, B). Whereas we had inferred direction following in earlier studies based on congruence of orientations and topographical features (Landler and Gollmann 2011; Landler et al. 2016), we had now refined the approach by recording the individual migration direction of each toad at the moment of encountering it. D-axis orientation might constitute a robust migratory behaviour in toads, which can be exploited for studies to investigate the underlying orientation mechanisms. Interestingly, d-axis orientation can switch from unimodal to bimodal orientation (along the same axis) from one experiment to the next. From the published and present data, it is difficult to assess what triggers either response.

Axial responses, however, are common in behavioural orientation studies (Malkemper et al. 2015, 2016; Muheim et al. 2016). They could indicate an underlying symmetrical compass system, e.g. axial symmetrical magnetic compass (Rodgers and Hore 2009; Winklhofer and Kirschvink 2010), or the involvement of axial sensitivity of neurons responding to directional cues (Jacob et al. 2016). Depending on the relative importance of the cues that animals use, they could easily switch between unimodal and axial responses. Slight differences in the presentation of orientation cues could alter the response, for example because one of the parameters is judged as not reliable by the animal’s neuronal navigational processing system.

In contrast, the toads did not orient towards their home pond. There are two possibilities to explain such a result: First, *B. bufo* might be unable to ‘truly’ navigate. Second, the method we used to explore ‘true navigation’ is unsuitable for this species.

One argument supporting the first possibility is that the resolution of a magnetic map might be 10 km at its best (Komolkin et al. 2017), therefore, not usable by an amphibian with a maximum of 3 km home range. However, there are other potential map cues available to the toads, for example olfactory cues. Odours might give the necessary precision (Wallraff 2004), especially when combined with beaconing in the vicinity to the goal (Joly and Miaud 1993). Several recent studies were unable to find homeward orientation in amphibians when displaced to very far and/or unfamiliar sites; this puts the ‘true navigation’ hypothesis in question (Pašukonis et al. 2014; Sinsch and Kirst 2015). However, the counter argument could be that animals need to be translocated even further, for a magnetic map mechanism to work, as the magnetic field changes are small with movements of only a few kilometres (Phillips 1996). This argument would pose the question of the biological relevance of such a navigation mechanism.

If the navigational abilities of the toads were limited, however, why are there many reports indicating surprising homing performances consistent with the use of map-like navigation systems (Heusser 1964, 1969; Sinsch 1987)? In order to give the animals enough time to position themselves on an internal map, we held toads for four days at the testing sites. Confining the toads to small containers for several days, however, may have compromised their motivation to show orientation behaviour in the arena (Landler et al. 2016). Nevertheless, at one site toads weakly oriented towards the d-axis on the second test day.

We collected toads from their way to the breeding pond, not directly out of the pond. Our rationale for collecting animals during their migration was that we surmised migrating toads to be highly motivated to reach the pond, whereas toads already present there might eventually lose the motivation to return later in the breeding season (Landler and Gollmann 2011). Perhaps some of the toads in our experiment attempted to orient towards the breeding pond, whereas others displayed direction following. The weak, but nearly significant, tendency of orientation towards the pond at site 2 (Fig. 3B, D) might indicate such a mixture of conflicting motivations, although this finding could also result from the fact that for some of the tested toads the homeward direction was similar to the d-axis (Fig. 2). Cloud cover and lunar cycle had small but significant effects on orientation in the arena (Table 2). As common toads often migrate on rainy nights, it is unlikely that they use a moon compass for orientation. Moon shadows, however, might influence their behaviour in trials in the arena. In view of the small effect sizes, we refrain from further speculations about possible causes of these findings. For future studies, we suggest to use tracking devices for orientation related research questions, as the fast-developing technologies in this area allow automated tracking and sampling of environmental variables as well as experimental manipulations (Guilford et al. 2011; Pašukonis et al. 2018).

Problems with replicability of research results are not restricted to navigation studies, but have triggered intense discussions of conceptual and statistical questions in the behavioural, biomedical and social sciences (Amrhein et al. 2019; Romero 2019). In empirical research, it is impossible to repeat an experiment exactly. In our case, the collection site of the toads was much closer to the pond than in the previous studies (due to the topography of the area); the various testing sites differed in many uncontrolled environmental parameters, some of which may have affected the cognitive abilities of the animals. For these reasons, one cannot expect perfect replication of the results, even if the same experimental protocol is followed carefully. A major cause of the “replicability crisis” is publication bias: experiments yielding statistically significant results are much more likely to become published than those that did not. Hence, scientists – in their roles as authors and editors – should consider conceptual and ethical arguments for publication of negative results (Mlinarić et al. 2017).

## Supplementary Material

R scripts

Table S1

Table S2

Supplementary Material

## Figures and Tables

**Table 1:** MANOVA table showing results from the first test day after AIC-based model selection. Degrees of freedom (df), Pillai test statistics (test statistics), approximated F statistics (approx. F), degrees of freedom for the numerator (num df), degrees of freedom for the denominator (den df), p-values (p) and effect sizes (eta^2^) are shown for selected model.

Factor	df	test statistics	approx F	num df	den df	p	eta^2^
**Cloud cover**	1	0.056	2.719	2	92	0.071	0.056
**Location**	1	0.060	2.975	2	92	0.056	0.061

**Table 2:** MANOVA table showing results from the second test day after AIC-based model selection. Degrees of freedom (df), Pillai test statistics (test statistics), approximated F statistics (approx. F), degrees of freedom for the numerator (num df), degrees of freedom for the denominator (den df), p-values (p) and effect sizes (eta^2^) are shown for selected model.

Factor	df	test statistics	approx F	num df	den df	p	eta^2^
**Cloud cover**	1	0.071	3.481	2	91	0.035	0.071
**cos_lunar**	1	0.090	4.524	2	91	0.013	0.090
**sin_lunar**	1	0.047	2.241	2	91	0.112	0.047
