# Supplementary material for: Orientation and navigation in Bufo bufo: a quest for repeatability of arena experiments
Source: Herpetozoa. Author manuscript; Available in PMC 2022 Apr 19. (PMC7612639; doi:10.3897/herpetozoa.33.e52854)
Supplement: Supplementary Material [file EMSEMS144319-supplement-Supplementary_Material.pdf]

Supplementary material 1

R-script 1. R code to reproduce analysis and plots

Authors: Markus Pail, Lukas Landler, Günter Gollmann
Data type: R file
Explanation note: Can be opened using R, open source and free statistical computing software. In order to run it supplementary materials 2, 3 and 4 have to be placed in the working directory.
Copyright notice: This dataset is made available under the Open Database License (http://opendatacommons.org/licenses/odbl/1.0/). The Open Database License (ODbL) is a license agreement intended to allow users to freely share, modify, and use this Dataset while maintaining this same freedom for others, provided that the original source and author(s) are credited.
Link: https://doi.org/10.3897/herpetozoa.33.e52854.suppl1

Supplementary material 2

Table S1. Raw data for the first test day

Authors: Markus Pail, Lukas Landler, Günter Gollmann
Data type: csv-file
Explanation note: This file is called when running supplementary material 1 in R.
Copyright notice: This dataset is made available under the Open Database License (http://opendatacommons.org/licenses/odbl/1.0/). The Open Database License (ODbL) is a license agreement intended to allow users to freely share, modify, and use this Dataset while maintaining this same freedom for others, provided that the original source and author(s) are credited.
Link: https://doi.org/10.3897/herpetozoa.33.e52854.suppl2

Supplementary material 3

Table S2. Raw data for the second test day

Authors: Markus Pail, Lukas Landler, Günter Gollmann
Data type: csv-file
Explanation note: This file is called when running supplementary material 1 in R.
Copyright notice: This dataset is made available under the Open Database License (http://opendatacommons.org/licenses/odbl/1.0/). The Open Database License (ODbL) is a license agreement intended to allow users to freely share, modify, and use this Dataset while maintaining this same freedom for others, provided that the original source and author(s) are credited.
Link: https://doi.org/10.3897/herpetozoa.33.e52854.suppl3

Supplementary material 4

R-script 2. Adapted circular functions not included in R packages

Authors: Markus Pail, Lukas Landler, Günter Gollmann
Data type: R file
Explanation note: This file is called when running supplementary material 1 in R.
Copyright notice: This dataset is made available under the Open Database License (http://opendatacommons.org/licenses/odbl/1.0/). The Open Database License (ODbL) is a license agreement intended to allow users to freely share, modify, and use this Dataset while maintaining this same freedom for others, provided that the original source and author(s) are credited.
Link: https://doi.org/10.3897/herpetozoa.33.e52854.suppl4
